# Adjuvant trastuzumab in the treatment of her-2-positive early breast cancer: a meta-analysis of published randomized trials

**DOI:** 10.1186/1471-2407-7-153

**Published:** 2007-08-08

**Authors:** Gustavo A Viani, Sergio L Afonso, Eduardo J Stefano, Ligia I De Fendi, Francisco V Soares

**Affiliations:** 1 Department of Radiation Oncology, Faculdade de Medicina de Marília, Faculty of Medicine of Marília (FAMEMA), Marília, São Paulo, Brazil

## Abstract

**Background:**

Breast cancer is the most common cancer in women in the U.S. and Western Europe. Amplification of the her-2/neu gene occurs in approximately 25% of invasive ductal carcinomas of the breast. The first HER-2/neu-targeted approach to reach the clinic was trastuzumab, a humanized monoclonal antibody directed against the extracellular domain of the HER-2/neu protein. Trastuzumab therapy prolongs the survival of patients with metastático HER-2/neu-overexpressing breast cancer when combined with chemotherapy and has recently been demonstrated to lead to dramatic improvements in disease-free survival when used in the adjuvant therapy setting in combination with or following chemotherapy. Here, we performed a meta-analysis of completed clinical trials of adjuvant trastuzumab in the adjuvant setting. Survival, recurrence, brain metastases, cardiotoxicity and directions for future research are discussed.

**Methods:**

A meta-analysis of randomized controlled trials (RCT) was performed comparing adjuvant trastuzumab treatment for HER2-positive early breast cancer (EBC) to observation. The MEDLINE, EMBASE, CANCERLIT and Cochrane Library databases, and abstracts published in the annual proceedings were systematically searched for evidence. Relevant reports were reviewed by two reviewers independently and the references from these reports were searched for additional trials, using guidelines set by QUOROM statement criteria.

**Results:**

Pooled results from that five randomized trials of adjuvant Trastuzumab showed a significant reduction of mortality (p < 0.00001), recurrence (p < 0.00001), metastases rates (p < 0.00001) and second tumors other than breast cancer (p = 0.007) as compared to no adjuvant Trastuzumab patients. There were more grade III or IV cardiac toxicity after trastuzumab (203/4555 = 4.5%) versus no trastuzumab (86/4562 = 1.8%). The likelihood of cardiac toxicity was 2.45-fold higher (95% CI 1.89 – 3.16) in trastuzumab arms, however that result was associated with heterogeneity. The likelihood of brain metastases was 1.82-fold higher (95% CI 1.16 – 2.85) in patients who received trastuzumab.

**Conclusion:**

The results from this meta-analysis are sufficiently compelling to consider 1 year of adjuvant trastuzumab treatment for women with HER-2-positive EBC based on the risk: benefit ratio demonstrated in these studies. Adequate assessment of HER-2/neu status is critical, and careful cardiac monitoring is warranted because of cardiac toxicity. Clinical trials should be designed to answer unsolved questions.

## Background

Breast cancer is the most common cancer in women in the U.S. and Western Europe. More than 210,000 women were predicted to be diagnosed, and more than 40,000 were predicted to die from the disease in 2005 in the U.S. [1]. HER-2/neu belongs to a family of four transmembrane receptor tyrosine kinases that mediate cell growth, differentiation, and survival [2,3]. Overexpression of the HER-2/neu protein, amplification of the her-2/neu gene, or both occurs in 20%–25% of breast cancers [4,5]. HER- 2/neu-positive breast cancer is an aggressive type that has a high rate of recurrence and short disease-free intervals after adjuvant (postoperative) chemotherapy [4]. Trastuzumab (Herceptin^®^; F. Hoffmann-La Roche Ltd., Basel, Switzerland), a monoclonal antibody directed against HER-2, improves survival and quality of life when given in combination with taxanes as first-line therapy in women with metastatic breast cancer [3-5] and has shown efficacy as monotherapy [6,7]. We now have the results of five large appropriately powered studies assessing the role of trastuzumab in addition to adjuvant chemotherapy for patients with HER2 positive tumours [5-7]. In this way, our group investigated whether the administration of trastuzumab was effective as adjuvant treatment for HER2-positive breast cancer if used after completion of the primary treatment for reducer mortality, recurrence, metastases and subsequent other tumors than breast cancer rate. Also, the another objective the current review was to evaluate the incidence of cardiac toxicity and brain metastases to give a more balanced view of the total evidence and to increase statistical precision.

## Methods

### Purpose

To investigate whether the administration of trastuzumab was effective as adjuvant treatment for HER2-positive breast cancer if used after completion of the primary treatment for mortality, recurrence, metastases and second tumor no breast cancer rate. Also, the another objective the current review was to evaluate the incidence of cardiac toxicity and brain metastases to give a more balanced view of the total evidence and to increase statistical precision.

### Types of studies, participants, interventions and outcome measures

Published randomized controlled trials were eligible for this metaanalysis. Published abstracts were included but unpublished studies were not sought. Studies published in any language were also eligible if they fulfilled the inclusion criteria. No authors were contacted for clarification or verification of patient data. All the trastuzumab adjuvant trials enrolled patients with HER-2-positive (immunohistochemistry 3+/fluorescence in situ hybridization positive or chromogenic in situ hybridization positive for FinHer) invasive breast cancer resected by lumpectomy or mastectomy. Patients could have node positive (all trials) or high-risk, node-negative (N9831, HERA, BCIRG 006, Fin her) disease, and all patients were to receive adjuvant chemotherapy and appropriate radiotherapy and hormonal therapy. Patients were required to have no locally advanced or distant disease and no previous or current cardiac disease. Trastuzumab administered i.v. at any dose and for any time of duration in the adjuvant setting. Differences in mortality, recurrence, Metastases, brain metastases, second tumor non breast cancer and cardiac toxicity rates (classified according to New York Heart Association) were collected. Mortality was defined as death from any cause, recurrence was defined as recurrence of breast cancer at any site; Metastases was defined as of the first distant tumor recurrence, ignoring locoregional recurrences and second breast or non breast cancers. Brain metastases were defined as recurrence in CNS. The term "other tumors no breast cancer" was used to define tumors that appeared after the treatment excluding new case of breast cancer or recurrences of that.

### Search strategy for identification of studies

Medline and manual searches were done (completed independently and in duplicate) to identify all published (manuscripts and abstracts) randomized controlled trials (RCTs) that comparing adjuvant trastuzumab treatment for HER2-positive early breast cancer to observation. The Medline search was done on PubMed between 1966 and 2006 with no language restrictions, using the search terms "trastuzumab," breast cancer" or "metastases," adjuvant trastuzumab" or "post operative traztusumab," and "her-2 overexpression." The second search was done through CancerLit, and the Cochrane Library to identify randomized trials published between January 1998 and July 2006, using MeSH headings (trastuzumab, adjuvant trastuzumab, her-2 over expression, breast cancer/sc {Secondary}, ex-lode Clinical Trials, clinical trial {publication type}) and text words (breast, cancer, adjuvant:, trastuzumab, trial, and study) without language restrictions. All the searched abstracts were screened for relevance. Manual searches were done by reviewing articles and abstracts cited in the reference lists of identified RCTs, by reviewing the first author's article, abstract file, from reference lists of retrieved papers, textbooks and review articles. Also, abstracts published in the Proceedings of the Annual Meetings of the American Society of Clinical Oncology (through 2005) were systematically searched for evidence relevant to this meta analysis. The selection of studies for inclusion was carried out independently by two of the authors (V-GA and S-LA). Each study was evaluated for quality using the scale of 1 to 5 proposed by Jadad [8]. If reviewers disagreed on the quality scores, discrepancies were identified and a consensus was reached. Trial data abstraction was also done independently and in duplicate, but abstractors were not blinded to the trials' authors or institution. The reviewers were familiar with the trials, and there were only five trials, so blinding the abstracts was not practical. Any discrepancies in data abstraction were examined further and resolved by consensus.

### Analysis of the review

The data analyses were made with Review Manager Version 4.2 provided by The Cochrane Collaboration. All analyses were carried out on an intention-to-treat basis; that is, all patients randomly assigned to a treatment group were included in the analyses according to the assigned treatment, irrespective of whether they received the treatment or were excluded from analysis by the investigators. For categorical variables, weighted risk ratios and their 95% confidence interval were calculated using RevMan 4.2 software according to the Peto method [22]. Results were tested for heterogeneity at significance level of P < 0.05 according to the methods outlined by Der Simonian and Laird [23]. A fixed effects model was used if there was no evidence of heterogeneity between studies, if there was evidence of heterogeneity random effects model was used for meta-analysis. The odds ratio and 95% confidence interval were calculated for each trial and presented in a Forrest plot. Sensitivity analyses was performed by excluding the trials which Jadad-scale was only 1 score. Publication bias is a common concern in meta-analysis that is related to the tendency of journals to favor the publication of large and positive studies. We chose a commonly used method for detecting publication bias, which is a graphical plot of estimates of the ORs from the individual studies versus the inverse of their variances, which is commonly referred to as a "funnel plot." An asymmetry in the funnel would be expected if there was publication bias with smaller studies tending to show larger ORs, because small studies with no significant statistical results would be less likely to be reported.

## Results

**Figure 1:**
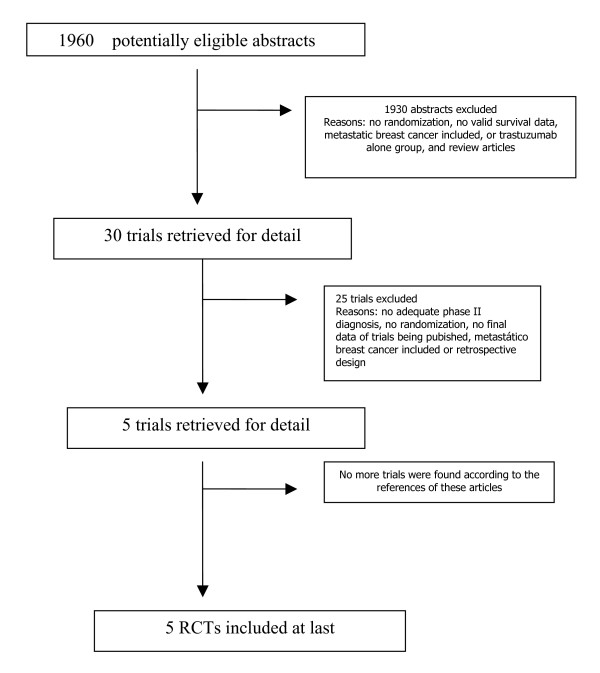
The flowchart. RT: radiotherapy; RCTs: randomized controlled trials, EBCT: Early Breast conserving therapy.

The two trial assessors agreed on the selection of five RCTs [11-14]. The Quorum flow diagram illustrates the main reasons for trial exclusion (Figure 1). Combining these trials yielded data on 9117 patients. Four large, randomized, clinical trials and one smaller randomized Finnish trial of trastuzumab as adjuvant therapy for operable breast cancer have been completed and their initial results reported: the Breast International Group (BIG) Herceptin^® ^Adjuvant (HERA) [12], Breast Cancer International Research Group (BCIRG)-006 [13], the NSABP B-31 [11], Intergroup N9831 [11], and Finnish FinHer trial [14] (Table 1). Two arms of those studies were not included in this review. One arm of the BIG HERA trial with 2-year of use of trastuzumab with 1694 patients was excluded. Another arm excluded was of the BCIRG-006 trial, which used docetaxel and carboplatin plus trastuzumab (TCH) as adjuvant systemic therapy. These arms of studies were excluded because had not group controls in other studies or in same them.

**Table 1:** Summary of patient characteristics from the trastuzumab adjuvant trials

**Characteristic **(%)	BCIRG006	NSABP/N9831	HERA	FIN HER
Median age	49	50	49	51
Age<50 years	52	51	51	51
Diameter primary tumor (%)				
Tumor >= 20 mm	58	59.6	48.5	64.5
Estrogen receptor status (%)				
Positive	56	52.2	54.5	47
Negative	44	47.6	45.5	53
Progesterone receptor status (%)				
Positive	38	40.3	48.5	34
Negative	62	59.3	61.5	66
Node negative disease	29	5.7	32	16
Grade III tumor	NA	69	60	65
Planed endocrine therapy	54	52	46	-
Taxane based chemotherapy	100	100	26	50
Quality trials by jadad scale	4	4	4	4

### Description of studies

The designs of all four major adjuvant trastuzumab trials and the smaller Fin Her study are summarized in Table 1 and 2. All the trastuzumab adjuvant trials enrolled patients with HER-2-positive (immunohistochemistry 3+/fluorescence in situ hybridization positive or chromogenic in situ hybridization positive for FinHer) invasive breast cancer resected by lumpectomy or mastectomy. Patients could have node positive (all trials) or high-risk, node-negative (N9831, HERA, BCIRG 006, FinHer) disease, and all patients were to receive adjuvant chemotherapy and appropriate radiotherapy and hormonal therapy. In addition, patients were required to have no locally advanced or distant disease and no previous or current cardiac disease. Cardiac eligibility criteria differed between the trials. The HERA trial required patients to have a normal left ventricular ejection fraction (LVEF) ≥ 55% (as measured by echocardiography or multiple-gated acquisition [MUGA] scan) after completion of chemotherapy and radiotherapy, while in the B-31 and N9831 trials, baseline LVEF was required to be ≥ 50% after completion of chemotherapy. In the BCIRG 006 trial, baseline LVEF was required to be ≥ 50% after surgery. Additional cardiac exclusion criteria included a history of myocardial infarction, congestive heart failure (CHF), coronary artery disease, angina pectoris requiring medication, uncontrolled hypertension, clinically significant valvular disease, or unstable arrhythmias.

**Table 2:** Summary of cardiac safety with trastuzumab in early breast cancer

**TRIAL**	**ARM**	**Baseline LEVF(%)**	**CHF(%)**	**Cardiac death**	**Cardiac follow-up**
**HERA**	NIL	>= 55	0	1	MUGA scan or echocardiogram at 3–4 wks prior to randomization, and 3, 6, 12, 18, 24, 30, 36, and 60 mos from randomization
	H 1 YEAR		0.6	0	
**NSABP-31**	AC followed P	>= 50	0.8	1	MUGA scan or echocardiogram 3 wks after last AC dose, 6, 9, and 18 mos from randomization, and 3 mos after last trastuzumab dose
	AC followed PH		4.1	0	
**NCCTGN9831**	AC followed P	>= 50	0.3	1	MUGA scan 3 wks after last AC dose, 6 and 9 mos from randomization, and 3 mos after the last trastuzumab dose
	AC followed P and H		2.5	1	
	AC followed PH		3.5	0	
**BCIRG006**	AC followed D	>= 50	0.3	0	After last AC dose, after second docetaxel dose, after end of chemotherapy, and at 3, 12 and 36 mos from randomization. At baseline, at 6 wks, 4.5 mos,13.5 mos, and 37.5 mos from randomization
	AC followed DH		1.6	0	
	DCarbo followed H		0.4	0	
**FIN HER**	NO H	>= 50	3	0	MUGA scan or echocardiogram before chemotherapy, after CEF, and 12 and 36 mos after completion of chemotherapy
	H		0	0	

AC, doxorubicin plus cyclophosphamide; BCIRG, Breast Cancer International ;NCCTG, North Central Cancer Treatment Group; NSABP, National Surgical Adjuvant Breast and Bowel Project; P, paclitaxel. MUGA, multigated acquisition;Research Group; Carbo, carboplatin;CHF, congestive heart failure; cum, cumulative incidence; D, docetaxel; H, trastuzumab; HERA, Herceptin^® ^Adjuvant; LVEF, left ventricular ejection fraction;

### NSABP-31 and N9831

The NSABP B-31 trial [11] compared four cycles of doxorubicin and cyclophosphamide (AC) followed by four cycles of every-3-week paclitaxel (P) (AC.P, arm1) with AC.P plus 52 weeks of trastuzumab (H) beginning with the first cycle of P (arm 2). The N9831 trial [11,13] was a three-arm study that compared four cycles of AC followed by 12 weekly doses of paclitaxel (AC.P, arm A) with AC.P followed by 52 weeks of H beginning after P (arm B) and with AC.P plus 52 weeks of H beginning with the first P cycle (arm C). Because arm 1 and arm 2 of the B-31 trial are similar to arms A and C of the N9831 trial, the studies were amended to include a joint statistical analysis combining arm 1 and arm A for comparison with arm 2 and arm C. Arm B was excluded from the analysis because H was not given concurrently with P. In both trials, only patients with tumors scored as with 3+ or more staining for HER2 by immunohistochemistry (IHC) or gene amplification by fluorescence in situ hybridization (FISH) were eligible. HER2 testing was centrally confirmed or performed in approved reference laboratories. In an intent-to treat analysis with a combined median follow-up of two years (2.4 years for B-31 and 1.5 years for N-9831), there was a highly significant 52 percent reduction in the risk of disease recurrence with sequential trastuzumab (three-year DFS 87 versus 75 percent, HR 0.48), and despite the short follow-up, a 33 percent reduction in the risk of death (three-year OS 94.3 versus 91.7 percent, HR 0.67) [11].

### Fin her

Patients enrolled in the smaller FinHer trial were randomized to three cycles of docetaxel or vinorelbine followed by three cycles of fluorouracil, epirubicin, and cyclophosphamide [14]. The primary aim of that trial was to compare treatment using docetaxel with treatment using vinorelbine. The subset of women with HER-2-positive tumors (n = 232) was further randomized to either receive or not receive trastuzumab for 9 weeks together with the first three cycles of docetaxel or vinorelbine [14]. The primary end point of FinHer was recurrence-free survival; secondary end points included adverse events (AEs), the effect of treatment on LVEF, time to distant recurrence, and overall survival [14]. Within this subgroup, DFS was significantly better among those who received trastuzumab (89 versus 78 percent, p = 0.01) and there was a trend toward better OS (96 versus 90 percent, p = 0.07). The magnitude of those benefits is similar to those seen in the other trastuzumab trials that used one or two years of treatment.

### HERA

The HERA trial [12] was an international, multicenter, randomized, controlled trial comparing 1 year or 2 years of trastuzumab given every 3 weeks with observation (no trastuzumab) in patients with HER-2/neu-positive early breast cancer who had completed locoregional therapy and at least four cycles of neoadjuvant or adjuvant chemotherapy from a list of approved regimens. Data were available for 1,694 patients assigned to 2 years of treatment with trastuzumab, 1,694 patients assigned to 1 year of trastuzumab, and 1,693 patients assigned to observation. The results of 1 year of trastuzumab versus observation (first planned interim analysis) were recently reported (n = 3,387) [12]. The most recent report included 1698 controls and 1703 patients treated with one year of trastuzumab, who were followed for an average of 24 months [12]. There was a statistically significant 36 percent reduction in disease recurrence (HR 0.64, three-year DFS of 81 versus 74 percent) as well as a significant improvement in overall survival (HR 0.66, 92 versus 90 percent in the trastuzumab and nontrastuzumab groups, respectively). There were twice as many grade 3 or 4 adverse events with trastuzumab (11 versus 6 percent). The only death attributed to cardiac causes was in the control arm. Trastuzumab was discontinued by 72 women (4 percent) because of cardiac problems.

### BCIRG 006

The BCIRG-006 trial [13] was limited to patients whose tumors had evidence of her-2/neu gene amplification using FISH and included both node-positive and node negative patients. This study evaluated the efficacy and safety of three regimens as adjuvant systemic therapy after surgery: (1) doxorubicin and cyclophosphamide followed by trastuzumab plus docetaxel chemotherapy (AC.TH), (2) docetaxel and carboplatin plus trastuzumab (TCH), and (3) AC followed by docetaxel alone (AC.T) as the control arm. The results of a planned interim analysis of 3,222 patients after approximately one third of the required number of relapses had occurred were recently presented [13]. In a preliminary report with 23 month median follow-up, DFS was significantly better in both trastuzumab arms as compared to AC followed by docetaxel (hazard ratio for DFS 0.49 and 0.61 for AC/docetaxel/trastuzumab and TCH, respectively) [13]. There was no significant difference between the two trastuzumab-containing arms. However, the absolute benefit in DFS from an anthracycline plus trastuzumab compared to TCH was 4 percent. There were fewer symptomatic cardiac events and a lower incidence of asymptomatic LVEF decline with TCH compared to either anthracycline group in terms of two endpoints.

### Methodological quality of included studies

Each published randomized trial reported was assessed for quality using the validated scale developed by Jadad et al [8]. All the studies included were randomized. All studies reported the randomization procedure. The quality scores of included studies are summarized in the table of characteristics, Table 1.

### Overall mortality

**Figure 2:**
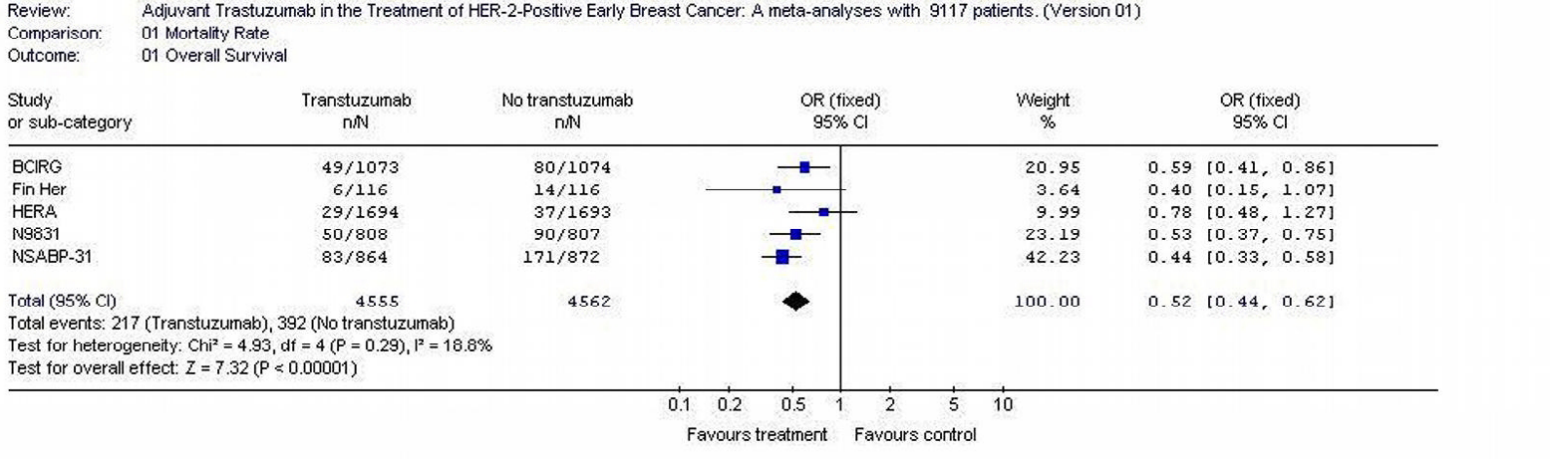
mortality rate of the adjuvant trastuzumab trials in early Breast Cancer.

All the studies reported overall survival as one of the outcomes. Altogether, the analyses included 5 trials with 9117 patients. The overall mortality rates was decreased for trastuzumab arm (217/4555 = 6%) compared to no trastuzumab arms (392/4562 = 8.5%). The individual odds ratios ranged from 0.44 to 0.78 with a pooled odds ratio for all of the trials of 0.52 with a 95% confidence interval of 0.44 to 0.62. The test for heterogeneity was not statistically significant with p value 0.28, which indicates that the pooling of the data was valid. The overall odds ratio suggests that there is difference between trastuzumab arms and no trastuzumab arms in terms of overall mortality rate with p value <0.0001. Trastuzumab arms were superior to no trastuzumab in decreased mortality rate in 5 studies, reaching statistical significance in 3 RCTs [11,13], (Figure 2).

### Recurrence rate

**Figure 3:**
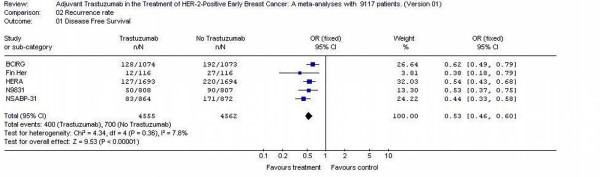
Recurrence rate of the adjuvant trastuzumab trials in early Breast Cancer.

Five studies [11-14] reported this outcome representing a total of 9117 patients. The disease free survival rates were 8.2% (400/4555) and 15.3% (700/4562) for trastuzumab arms and no trastuzumab arms, respectively. The individual odds ratios varied from 0.32 to 0, 68. The test for heterogeneity was not statistically significant (p = 0.36) allowing the results to be pooled. The overall odds ratio was 0, 53 (95% CI 0.46 – 0.60) which suggests that there was difference for disease free survival between the trastuzumab and no trastuzumab arms for adjuvant systemic therapy (Figure 3).

### Cardiac toxicity

**Figure 4:**
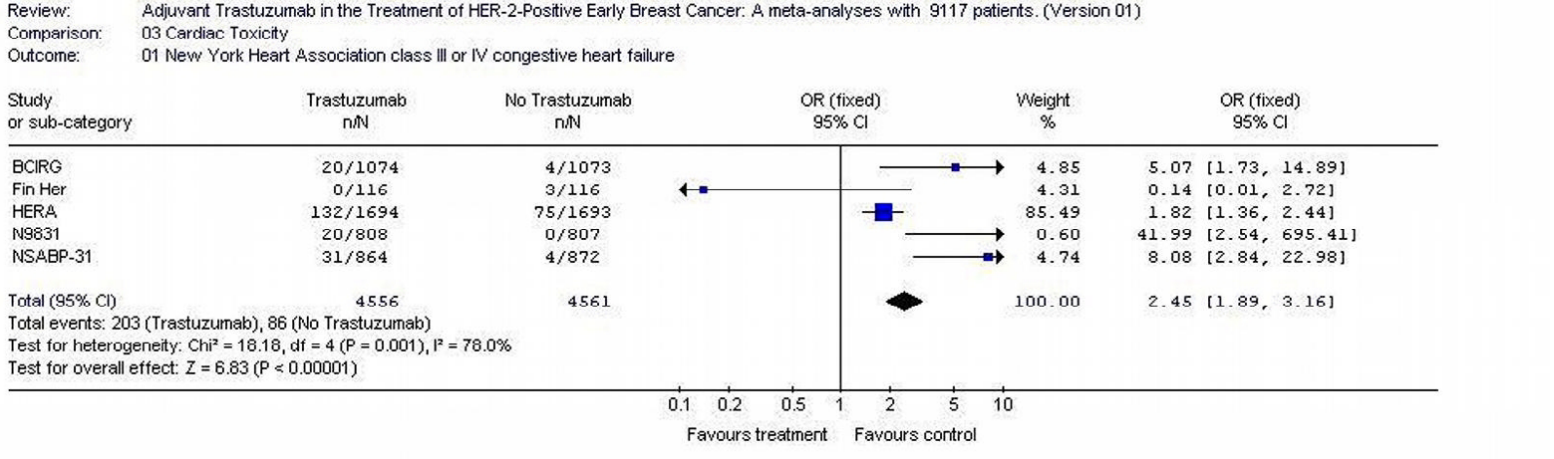
cardiac toxicity of the adjuvant trastuzumab trials in early Breast Cancer.

Five studies [11-14] had reported cardiac toxicity data and 9117 patients were included in the analysis. There were more cardiacs toxicity grade III or IV after trastuzumab use (203/4555 = 4.5%) compared to no trastuzumab (86/4562 = 1.8%). The likelihood of cardiac toxicity was 2.45-fold higher (95% CI 1.89 – 3.16) in trastuzumab arms patients. Test for heterogeneity was significant with p value of 0.001. Repeated analyses of the above end points excluding the fin her patient's only or using random effect did not alter the results and conclusion (Figure 4).

### Metastases rates

**Figure 5:**
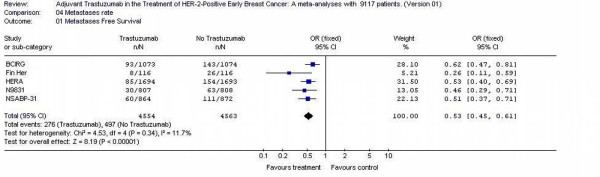
Metastases rate of the adjuvant trastuzumab trials in early Breast Cancer.

Five studies [11-14] reported the metastases rates. Nine thousand, one hundred and seventeen patients were randomized in those five studies. The metastases rates for all randomized patients were different; 6% (276/4555) for trastuzumab patients and 10.8% (497/4562) for no trastuzumab patients (p <0.00001). Tests for heterogeneity in the analysis were not significant (p = 0.34) (Figure 5).

### Brain metastases

**Figure 6:**
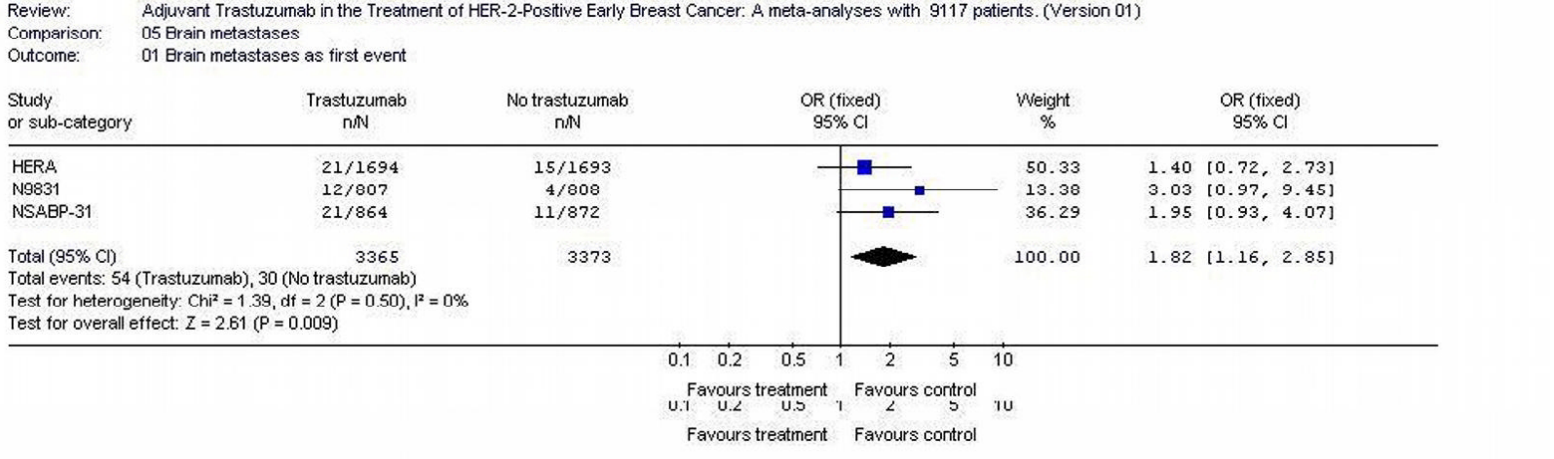
Brain metastases of the adjuvant trastuzumab trials in early Breast Cancer.

Three studies with 6738 patients reported brain metastases incidence rate. The likelihood of brain metastases was 1.82-fold higher (95% CI 1.16 – 2.85) in trastuzumab arms patients. Test for heterogeneity was not significant with p value of 0.50. (Figure 6)

### Second non breast malignancy

**Figure 7:**
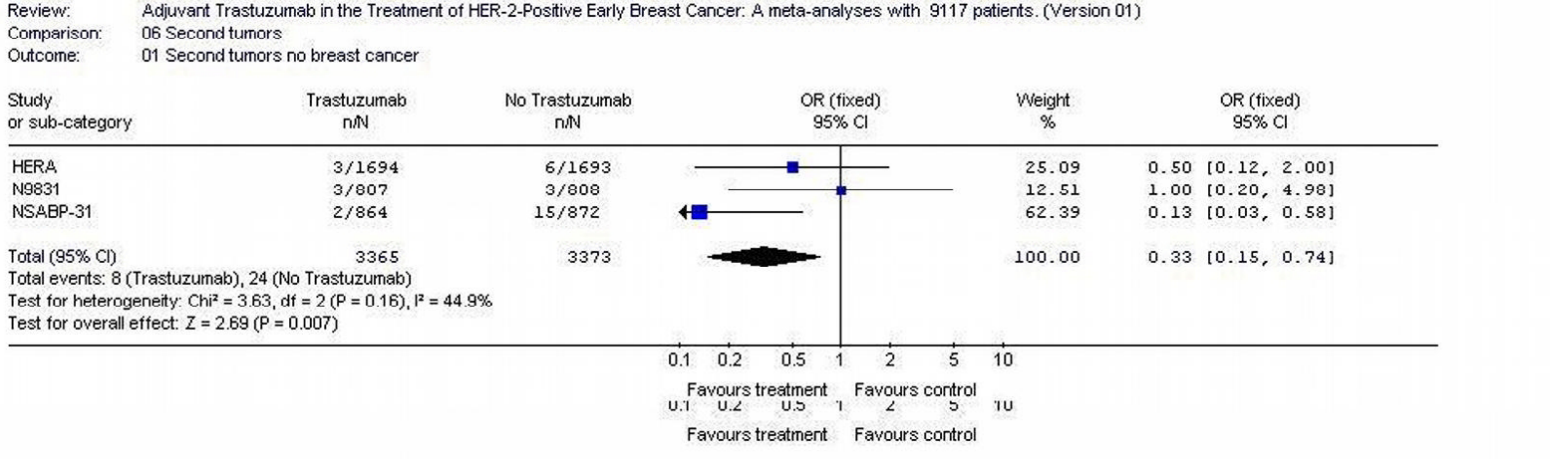
second non breast malignancy of the adjuvant trastuzumab trials in early Breast Cancer.

Only three studies [11,12] reported the second non -breast cancer malignancy, 6738 patients were randomized in those three studies. The likelihood of subsequent other tumors than breast cancer were 0.33-fold smaller (95% CI 0, 15 – 0.74) in trastuzumab arms patients. Test for heterogeneity was not significant with p value of 0.16 (Figure 7).

### Evaluation of publication bias

**Figure 8:**
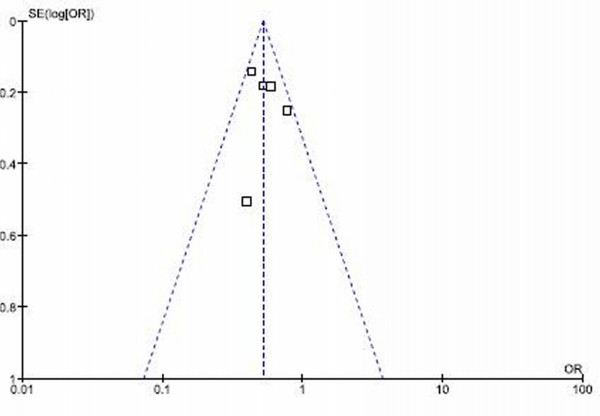
funnel plot for mortality rate in adjuvant trastuzumab trials.

The funnel plot of the log ORs versus the inverse of their variances of the individual studies is displayed in Figure 8. The plot formed a very distinct funnel shape with the log ORs evenly distributed around the meta-analysis OR regardless of the study variance. Therefore, there was no indication of an asymmetry in the study findings by the variance or size of the studies and, thus, little evidence for publication bias.

## Discussion

Data from our meta-analyses confirm that the addition of one year of trastuzumab to anthracycline and taxane-containing adjuvant chemotherapy regimens provides substantial benefit for women with HER2-positive breast cancer, both in terms of disease recurrence and survival. The results from all of the trials incorporating one year of trastuzumab into the adjuvant regimen demonstrate a statistically significant increase in the incidence of symptomatic cardiac dysfunction and asymptomatic decreases in LVEF. Trastuzumab was first employed for metastatic breast cancer (MBC). The activity and safety of trastuzumab as a single agent were investigated in two phase II clinical trials in women with HER2-overexpressing MBC who had progressed after one or two chemotherapeutic regimens [15,16]. The objective response rates were 11.6% and 15% in these two studies [15,16]. Cardiac dysfunction was the most common adverse event, occurring in 5% of treated patients, many of whom had received doxorubicin prior to trastuzumab. In a study that was conducted to investigate the efficacy and safety of trastuzumab as a single agent in the first-line treatment of HER2-overexpressing MBC, the response rate was 26% [17]. In that randomized phase II trial of first-line treatment, the patients were randomly assigned to one of two dose levels of trastuzumab (4 mg/kg initially followed by 2 mg/kg weekly, or 8 mg/kg to start, followed by 4 mg/kg weekly). The overall RR was 35% for women with 3+immunohistochemical (IHC) staining nearly double that reported for the previously treated patients. On the other hand, the overall RR was zero for those with 2+ IHC staining [17]. This study concluded that single agent trastuzumab was active and well-tolerated as a first-line treatment for women with MBC with HER2 3+ overexpression, determined by either IHC or gene amplification by FISH [17]. From these clinical trials, it has been shown that trastuzumab administered as a single agent is both active and well tolerated [15-17]. The trastuzumab adjuvant trials have also provided valuable information on the efficacy and safety of combining trastuzumab with specific standard chemotherapy regimens. The NSABP B-31 and NCCTG N9831 trials have shown that trastuzumab can be combined with a standard AC-paclitaxel regimen, and the BCIRG 006 trial has shown that trastuzumab can be combined with a standard AC-docetaxel and a nonanthracycline regimen. Furthermore, there was a significantly longer overall survival time with 1 year of adjuvant Trastuzumab in the BCIR 006, B-31, N9831 and a clear trend in the other studies. Ours results based in these three and others two large powered studies assessing the role of Trastuzumab in addition to adjuvant chemotherapy for patients with HER2 positive tumors in reduced the mortality rate, recurrence and second tumors other that breast cancer. Together, these data present a variety of effective Trastuzumab-based treatment options for clinicians.

### Brain metastases

Another question observed in our review was the high incidence of brain metastases in patients who received adjuvant Trastuzumab. The causes of these trends are unknown. Bendell and colleagues [20] retrospectively studied 122 women treated with trastuzumab alone or in combination with chemotherapy for Her-2-overexpressing metastatic breast cancer. Based on a median follow-up of 23 months, 34% of patients were diagnosed with CNS metastases, well above historical rates. At the time of diagnosis of CNS metastasis, 50% of patients were responding to therapy or had stable disease. That report was confirmed by the study of Clayton and colleagues [21] which followed 93 metastatic breast cancer patients. Brain metastases occurred in 25% of patients during a median follow-up period of 10.8 months from the start of trastuzumab therapy. One theory suggests that Her-2 overexpression endows tumor cells with increased metastatic aggressiveness to sites such as the lungs and may similarly augment metastatic propensity to the CNS [22,23]. Second, by allowing patients to live longer, trastuzumab may allow micrometastatic brain metastases to become symptomatic as a natural consequence of an extended life span. A nonexclusive, third theory posits that trastuzumab is effective against systemic metastases but relatively ineffective against CNS metastases due to its poor penetration of the blood-brain- barrier. That hypothesis may extend to cytotoxic chemotherapy as well as Trastuzumab [24]. Limited pharmacokinetic data in support of this hypothesis suggest that systemic administration of trastuzumab results in drug levels in the CSF that are 300-fold lower than in the serum [25]. Also, intrathecal administration of 4D5, the murine precursor of trastuzumab, shows efficacy against a human xenograft of Her-2-overexpressing cancer growing in the leptomeninges, suggesting that trastuzumab could be efficacious if it could penetrate the blood-brain- barrier [26]. Based on our data and other studies [20,21] we predict that brain metastases will become more prevalent as greater control over systemic metastases is achieved, particularly with regard to Her-2-positive tumors.

### Cardiac toxicity

The incidence of cardiac events with trastuzumab in the EBC setting remained at an acceptable level and was similar across the adjuvant trials, with a reported overall incidence that was 0.6%–3.3% higher [11-14]. Our results analyzing 9117 patients submitted to adjuvant trastuzumab by breast cancer suggest that sequencing the combination of trastuzumab and taxanes plus anthracycline-based chemotherapy increases the risk for class III or IV CHF (OR = 2.45, CI 95 1.89–3.16) However, in our analyses that result was associated with heterogeneity using random effect. Probably due to different definitions of cardiac events, evaluations for cardiac safety, analysis of cardiac end points, and duration of follow-up differed among the adjuvant trials and thus make comprehensive cross-trial conclusions difficult, as showed in Table 2. Comparisons regarding cardiac safety should therefore be approached with caution. Increasing evidence suggests that, in patients with metastatic breast cancer who experience a significant LVEF decline, management with beta-blockade and angiotensin-converting enzyme inhibition may allow careful reinitiation of trastuzumab therapy after assessment of the risk-benefit ratio [27]. In the B-31 trial, 27 of the 31 patients in the trastuzumab arm have been followed for>6 months after diagnosis of a cardiac event; 26 were asymptomatic at last assessment and 18 remained on cardiac medication [11]. In this way, the cardiac function of all patients who start treatment with trastuzumab should be monitored and with further follow-up, it will become clearer whether sequential or anthracycline-free regimens carry a lower risk of cardiac toxicity with equivalent efficacy. The mechanism of cardiac dysfunction associated with trastuzumab is not clearly understood, although several hypotheses have been proposed. These include the modification of anthracycline-induced cardiotoxicity, immunemediated destruction of cardiomyocytes, the effects on HER2 signaling pathways that are required for the maintenance of normal cardiac contractility, and the dependence on HER2 for myocyte survival, which is then impaired during trastuzumab treatment [27]. There is increasing experimental evidence supporting a direct toxic effect of HER2 blockade on the heart. HER2 signaling appears to play an important role in embryonic cardiac development and cardioprotection, at least in rodents [28,29]. Those data suggest that trastuzumab-related cardiotoxicity is not immune-mediated or due to effects outside the heart, and it does not result solely from the modification of anthracycline-induced cardiac toxicity. In contrast to anthracycline-related cardiotoxicity, trastuzumab-associated toxicity does not appear to be dose related, and it usually responds to standard medical treatment or the discontinuation of trastuzumab [30,31]. Thus, in the adjuvant setting, continued treatment with trastuzumab is contraindicated if there is any evidence of cardiac dysfunction, and monitoring for early evidence of left ventricular dysfunction is important. In contrast, continuation of trastuzumab therapy or resumption of treatment after resolution of cardiac abnormalities may be safe in some women with metastatic breast cancer who develop early evidence of cardiac dysfunction.

### Unresolved questions

The optimal timing of initiation of trastuzumab in patients with early-stage HER-2/neu-positive breast cancer who have completed adjuvant chemotherapy also has not been defined. The HERA trial allowed patients to begin trastuzumab up to 6 months after local treatment and chemotherapy had been completed. Whether trastuzumab would benefit patients who have completed chemotherapy >6 months previously is unknown. However, in patients with HER-2/neu-positive breast cancer treated with AC followed by paclitaxel chemotherapy, the hazard rate for recurrence increased significantly the first year and remained high in the second year after surgery. This would suggest that trastuzumab therapy is beneficial, although this situation has not been tested in randomized clinical trials. While the sequential administration of trastuzumab after chemotherapy appears to be effective, longer follow- up is needed to determine whether simultaneous and sequential administration with other chemotherapy agents are equally effective and whether there is a population of patients in whom chemotherapy is not necessary (patients with estrogen receptor-positive and HER-2/neu-positive disease, patients with negative lymph nodes and tumors < 1 cm) and for whom trastuzumab alone might represent appropriate adjuvant therapy. Patients in the NSABP B-31, N9831, and BCIRG-006 trials received adjuvant radiation upon completion of chemotherapy during maintenance trastuzumab. Clinical trials should be designed to answer those questions.

### Limitations of our study

One limitation in our study is the data source extracted from abstracted data or published studies and not individual patient data (IPD). Meta-analyses based on published data tend to overestimate treatment effects compared with individual patient data analyses. However, analyses using individual patient data may include fewer studies if all authors do not agree to submit their full databases to the analyzing group. In general, an IPD-based meta-analysis would give a more robust estimation for the association; therefore, we should interpret the results with care, especially for a positive result. Although the risk of publication bias exists in any meta-analysis, whether based on individual patient data or not, we feel that this was not an important aspect of our study, as many positive and negative trials were included in the analysis.

## Conclusion

The results of this metanalysis indicate that the addition of at least one year of trastuzumab to anthracycline and taxane-containing chemotherapy provides substantial benefit for women with HER2-positive breast cancer, both in terms of disease recurrence and survival. Trastuzumab has been approved for use in the adjuvant setting in the United States for women with node-positive, HER2-overexpressing breast cancer as part of a treatment regimen containing doxorubicin, cyclophosphamide, and paclitaxel. Although the data are insufficient to conclude whether sequential or concurrent administration is superior, cardiac toxicity may be worse with the concurrent approach. Despite close monitoring and aggressive management, combined treatment is associated with a small but real increase in the risk of myocardial dysfunction. In this way, patients must be screened for heart function before, during trastuzumab therapy and the risk of cardiotoxicity must be balanced against the poor prognosis of women with HER2-positive node-positive breast cancer. Adequate assessment of HER-2/neu status is critical, and clinical trials should be designed to answer unsolved questions.

## Competing interests

The author(s) declare that they have no competing interests.

## Authors' contributions

VG carried out the search, acquisition and interpretation of the data in studies. He also drafted the manuscript. VG performed the statistical analysis and drafted the manuscript. S LA participated in the design of the study; SE carried out the search for articles and gave final approval of the version to be published. and S FV participated in the design of the study; D LI gave final approval of the version to be published. All authors read and approved the final manuscript.

## Pre-publication history

The pre-publication history for this paper can be accessed here:


